# Enhanced removal of indigo carmine dye from aqueous solutions using polyaniline modified partially reduced graphene oxide composite

**DOI:** 10.1038/s41598-025-98115-8

**Published:** 2025-05-03

**Authors:** Saadia M. Waly, Ahmad M. El-Wakil, Mohamed M. Waly, Weam M. Abou El-Maaty, Fathi S. Awad

**Affiliations:** 1 Chemistry Department, Faculty of Science, Mansoura University, Mansoura, 35516 Egypt; 2 Chemistry Department, Faculty of Science, New Mansoura University, New Mansoura, 35712 Egypt

**Keywords:** Polyaniline, Graphene oxide, Indigo carmine, Remediation, Wastewater, Pollution remediation, Environmental chemistry

## Abstract

In this study, graphene oxide (GO) nanosheets were chemically modified by attaching polyaniline (PAN) nanoparticles to their surfaces, creating a polyaniline partially reduced graphene oxide composite (PAN@PRGO). This synthesized PAN@PRGO nanocomposite serves as an innovative and highly effective adsorbent for removing indigo carmine (IC) dye from water. The morphology and chemical composition of PAN@PRGO were analyzed using various techniques, including scanning electron microscopy (SEM), Fourier-transform infrared spectroscopy (FTIR), X-ray diffraction (XRD), and X-ray photoelectron spectroscopy (XPS), confirming the successful grafting of PAN onto the GO surface. Batch adsorption tests showed that PAN@PRGO has an outstanding adsorption capacity for indigo carmine (IC) dye, achieving 490.0 mg g^−1^ at pH 5.0 and 298 K. This is notably higher than the adsorption capacity of GO nanosheets alone (317.25 mg g^−1^) and exceeds that of other materials reported in the literature. Additionally, PAN@PRGO demonstrated 100% removal efficiency for IC dye at concentrations up to 300 mg L^−1^. The experimental data closely matched the Langmuir isotherm model and the pseudo-second-order kinetic model, suggesting that electron-sharing interactions between IC dye and PAN@PRGO contribute to the adsorption mechanism. The adsorbed IC dye was recoverable using a 0.1 M NaOH solution, with the composite retaining near-100% efficiency even after five adsorption–desorption cycles. These results indicate that the PAN@PRGO composite is a promising, reusable adsorbent for effective IC dye removal from industrial wastewater.

## Introduction

The growing concerns over public health necessitate ensuring a clean water supply, as access to safe and adequate water is crucial for reducing disease burdens and improving well-being^1–3^. Unfortunately, the expansion of industries such as textiles, plastics, printing, paper, and leather has resulted in the discharge of large volumes of wastewater contaminated with hazardous organic dyes. These dyes pose significant risks to both human health and the environment, making their removal from industrial effluents a pressing challenge^4–6^.

Indigo Carmine (IC) is a water-soluble anionic dye with a molar absorption coefficient of approximately 2 × 10^4^ M^−1^ cm^−1^ at 600 nm^7,8^. Due to its extensive use in textiles, pharmaceuticals, cosmetics, medicine, and the food industry, IC is frequently detected in wastewater, particularly from textile effluents. Even at trace levels, IC can alter water color, degrade water quality, and render it unfit for consumption^7,8^. Studies have reported IC concentrations in wastewater ranging from a few milligrams per liter to several hundred milligrams per liter, depending on the industrial source and treatment methods employed. For instance, IC concentrations of approximately 64.6 mg L^−1^ have been detected in textile wastewater, while levels as high as 500 mg L^−1^ and 200 mg L^−1^ have been reported in effluents from textile and dye manufacturing plants, respectively^9–12^.

Several methods have been explored for dye removal, including electrochemical oxidation, ion exchange, ozonation, biological treatment, photocatalytic degradation, membrane filtration, and adsorption^13–18^. Among these, adsorption is widely regarded as a promising approach due to its simplicity, cost-effectiveness, high removal efficiency, and ability to regenerate adsorbents for multiple cycles^13,19,20^. Various adsorbents, such as activated carbon^21^, polymers^22^, hydrogels^23^, metal oxides^24^, carbon nanotubes^25^, and metal–organic frameworks^26^, have been investigated for IC removal. In particular, carbon-based materials, including graphene oxide (GO) and its derivatives, have gained considerable attention due to their high surface area, chemical versatility, and superior adsorption capabilities^20,27,28^. GO, a two-dimensional nanomaterial with a unique layered structure, is rich in oxygen-containing functional groups (–OH, –COOH, –C=O, and epoxy), which facilitate electrostatic interactions, hydrogen bonding, and π–π stacking with dye molecules. While GO exhibits excellent adsorption properties, its strong hydrophilicity, structural instability, and tendency to aggregate in aqueous media can limit its effective surface area and hinder its reusability. Therefore, modifying GO to enhance its adsorption efficiency, selectivity, and long-term stability is essential for practical wastewater treatment applications^20,27–29^.

Among potential modifiers, polyaniline (PAN) has emerged as a promising candidate due to its unique electronic properties, ease of synthesis, and strong interactions with dye molecules via hydrogen bonding, electrostatic forces, and π–π interactions^30^. PAN modification of GO addresses key limitations associated with unmodified GO by improving its dispersion, preventing nanosheet restacking, and introducing additional nitrogen-containing functional groups (–NH, =N–, –NH_2_), which further enhance dye adsorption^31,32^. Despite several studies on polyaniline grapheme oxide composites (PAN-GO) for dye removal, most research has focused on general dye adsorption rather than the selective removal of IC. Thus, there remains a research gap in optimizing PAN-GO composite for IC removal from wastewater.

**Fig. 1:**
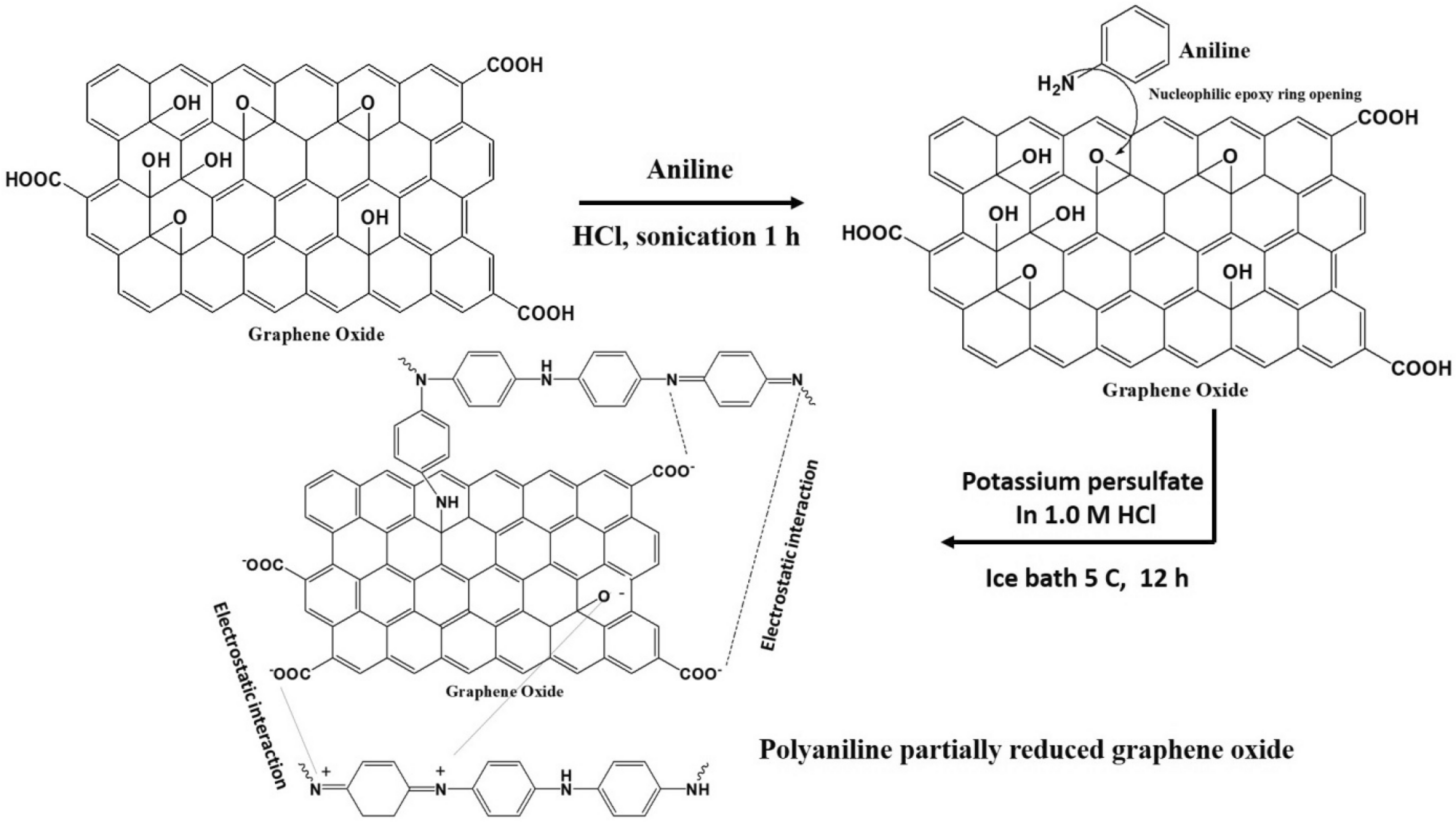
The general procedure for the preparation of PAN@PRGO composite.

In this study, a polyaniline partially reduced graphene oxide (PAN@PRGO) composite was synthesized via in situ polymerization of aniline on GO nanosheets through π–π stacking, electrostatic interactions, and hydrogen bonding. The synergy between PAN and partially reduced GO (PRGO) enhances the composite’s adsorption kinetics, stability, and reusability. Comprehensive characterization using SEM, FTIR, XPS, and XRD was conducted to confirm successful composite formation. The adsorption performance of PAN@PRGO for IC dye was systematically evaluated under various conditions, including initial dye concentration, pH, contact time, adsorbent dosage, temperature, and reusability. To ensure the practical relevance of this study, IC concentrations ranging from 10 to 800 mg L^−1^ were selected, covering the typical contamination levels observed in real-world wastewater scenarios. This study provides a detailed investigation of PAN@PRGO composites as an advanced adsorbent for IC removal, contributing to the development of efficient and sustainable wastewater treatment technologies. Figure 1 presents an overview of the PAN@PRGO synthesis process.

## Experimental section

### Materials

High-purity graphite powder (99.999%) was utilized in this experiment. The oxidizing agents included concentrated sulfuric acid (H_2_SO_4_, 98%), phosphoric acid (H₃PO_4_, 99%), and potassium permanganate (KMnO_4_, 99%). Other chemicals involved were hydrogen peroxide (H_2_O_2_, 30%), ethanol (99%), potassium persulfate (K_2_S_2_O_8_, 99.9%), aniline (99.55%), and deionized water (DI). All chemicals were purchased from Sigma Chemical Co. For additional details on characterization, refer to the Supporting Information (S1).

### Synthesis of polyaniline partially reduced graphene oxide (PAN@PRGO) composite

Graphene oxide (GO) was synthesized following the procedure outlined in Ref.^2^. Improved GO (GO) was prepared by mixing a 1:9 concentrated H₃PO_4_/H_2_SO_4_ solution (60:540 mL) with a blend of KMnO_4_ (27.0 g) and graphite flakes (4.5 g) while maintaining the temperature below 30 °C in an ice bath. The reaction mixture was then heated to 50 °C and stirred for 12 h, followed by cooling to room temperature. Afterward, 30% H_2_O_2_ (4.5 mL) and 600 mL of iced distilled water were added. The mixture was centrifuged, and the solid product was washed sequentially with 200 mL each of deionized water, 30% nitric acid (HNO₃), and 2% ethanol. The final product was dried in a vacuum oven at 80 °C overnight to yield GO. For PAN@PRGO preparation, 0.2 g of IGO was dispersed in 75.0 mL of DI water and sonicated to create a stable suspension. Aniline (1.3 mL) was added to the suspension, followed by 1-h sonication. Next, 7.0 mL of concentrated HCl was introduced under continuous stirring for an additional hour. Then, 2.5 g of potassium persulfate dissolved in 1.0 M HCl was added while maintaining the reaction mixture at 50 °C in an ice bath with stirring for 12 h. After centrifugation, the finished product was rinsed with ethanol and DI water until the filtrate was clear, and it was then dried in an oven at 80 °C.

### Batch adsorption studies

Batch adsorption experiments for IC dye removal were conducted by stirring 10 mg of the PAN@PRGO composite as an adsorbent in 10 mL of IC dye solution inside a 20-mL glass vial under various conditions: IC concentrations (10–800 mg L^−1^), pH values (2–8), contact time (15–240 min), adsorbent dosage (5–30 mg), and temperatures (25–60 °C), all at a constant stirring rate of 250 rpm. Regeneration of the adsorbent was achieved using 0.1 M NaOH as a desorbing agent, and the recovered adsorbent was subsequently reused in further adsorption experiments. After each adsorption experiment, IC dye concentration was measured spectrophotometrically at ƛ = 600 nm. The adsorption capacity of PAN@PRGO and the amount of dye removed were determined using the following Eqs. (1) and (2)^33^:

1 $${\text{q}}_{\text{e }}= \frac{\left({\text{C}}_{\text{o}}-{\text{C}}_{\text{e}}\right)\text{V}}{\text{m}},$$

2 $$\text{\% E}= \frac{\left({\text{C}}_{\text{o}}-{\text{C}}_{\text{e}}\right)}{{\text{C}}_{\text{o}}}\times 100,$$

where C_i_ represents the initial concentration of IC (mg L^−1^), C_e_ denotes the equilibrium concentration of IC (mg L^−1^), V stands for solution volume (L), m for adsorbent dosage (g), and qe for the adsorbent’s absorption capability (mg g^−1^).

## Results and discussion

### Characterization of GO and PAN@PRGO composite

**Fig. 2:**
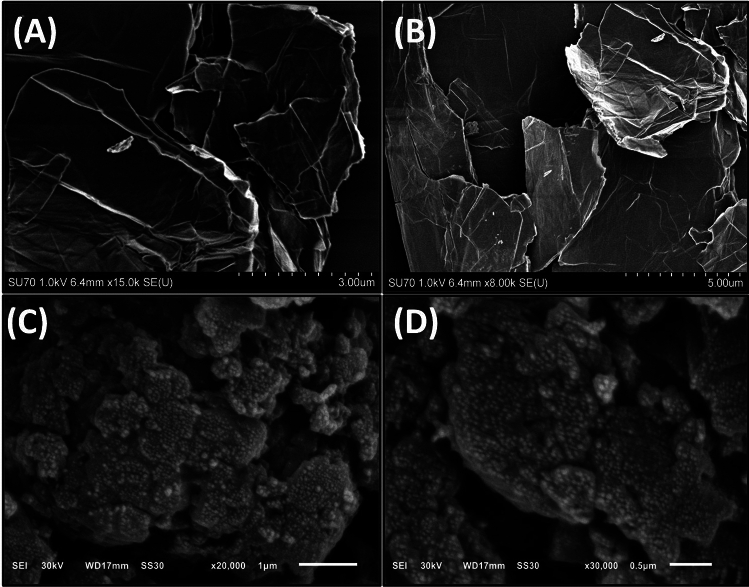
SEM images of GO (**A,B**), and PAN@PRGO (**C,D**).

The surface morphology of GO and PAN@PRGO was investigated using scanning electron microscopy (SEM). Figure 2 presents SEM images of GO and PAN@PRGO, revealing distinct morphological characteristics. GO exhibits an irregular, layered structure with uniformly stacked graphene nanosheets that possess a clean and smooth surface. In contrast, the SEM images of the PAN@PRGO nanocomposite indicate the successful formation of PAN nanoparticles, which are homogeneously distributed on the surface of the graphene nanosheets.

At higher magnifications, SEM images further reveal that PAN nanoparticles are spherical with well-defined sharp edges. Notably, the PAN matrix plays a crucial role in preventing GO sheet aggregation by acting as a spacer. This structural modification significantly enhances the effective surface area and increases the availability of active adsorption sites. Consequently, the improved morphology of PAN@PRGO contributes to enhanced adsorption efficiency. These findings confirm the successful grafting and in situ polymerization of PAN onto the surface of graphene oxide nanosheets, further validating the structural integrity and functionality of the synthesized composite.

**Fig. 3:**
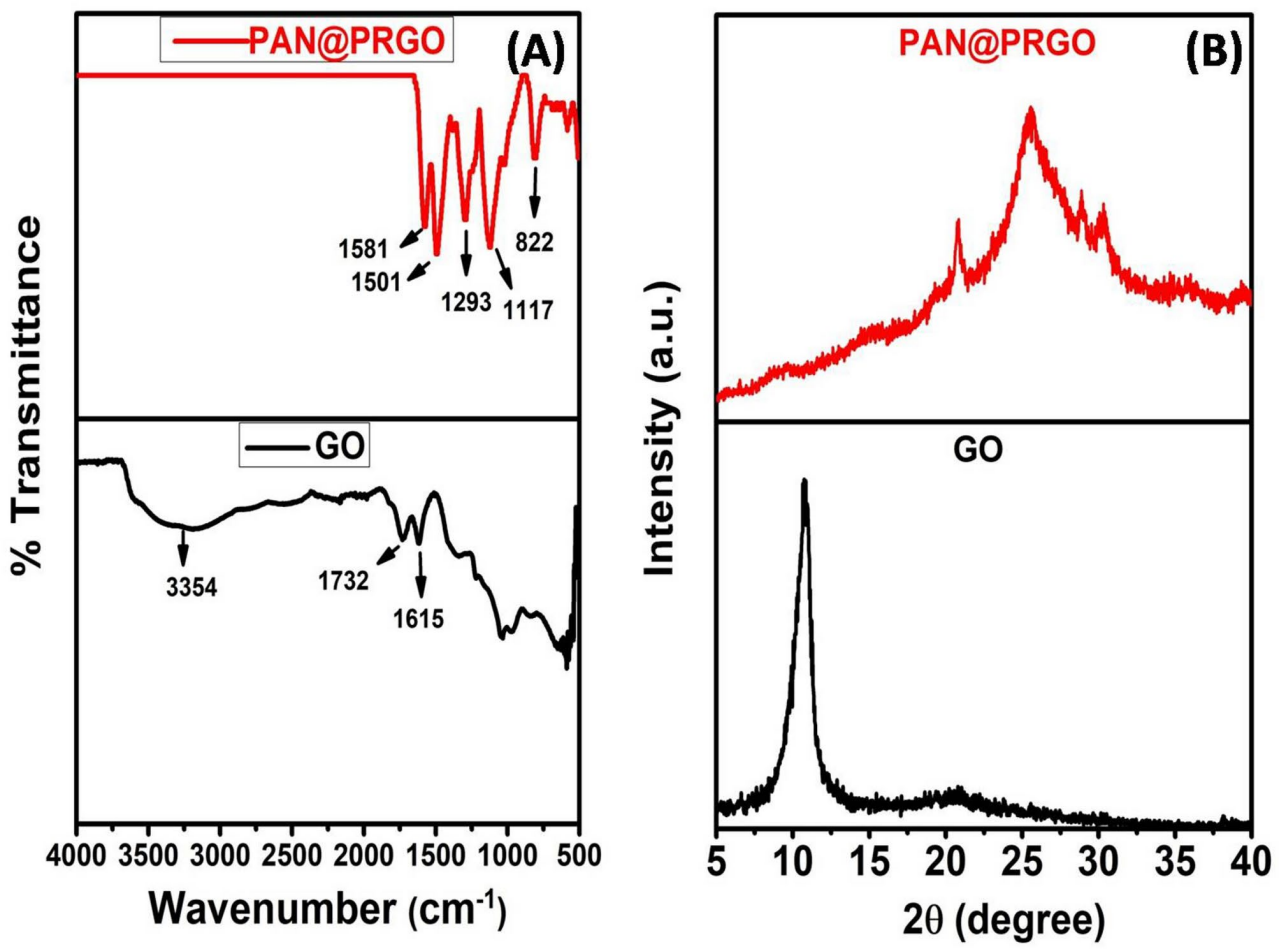
(**A**) FTIR spectra of GO, and PAN@PRGO. (**B**) XRD of GO and PAN@PRGO composite.

FTIR spectroscopy was used to examine the surface functional groups present in GO and the PAN@PRGO nanocomposite, as illustrated in Fig. 3A. The FTIR spectrum of PAN@PRGO displayed several distinctive new peaks not observed in GO alone. The peak at 1581 cm^−1^ is associated with the C=N bond vibration, while the 1501 cm^−1^ peak corresponds to the C=C bond vibration, which is linked to the benzene ring in polyaniline^34^. The peaks at 1293 cm^−1^ and 1117 cm^−1^ are attributed to the C–N stretching vibration of an aromatic secondary amine and the C=N stretching vibration, respectively^34–36^. The reduced intensity of the epoxy peak further supports the partial reduction of GO^37^. These observations confirm the successful attachment of PAN to the GO nanosheets through electrostatic interactions between the oxygen-containing functional groups on GO and the –C=  + N– groups in PAN^33,35,37^.

X-ray diffraction (XRD) analysis was conducted to characterize the crystalline structure of GO and the PAN@PRGO nanocomposite. In Fig. 3B, the GO pattern shows a strong, sharp peak at 10.8°, corresponding to an interlayer spacing that confirms its nanosheet structure. In contrast, the XRD pattern of PAN@PRGO shows the disappearance of the GO peak at 2θ = 10.8°, indicating the partial reduction of GO functional groups during in-situ polymerization. Four new characteristic peaks at 2θ values of 20.76°, 25.4°, 28.8°, and 30.3°, with respective interlayer spacings of 4.273 Å, 3.503 Å, 3.091 Å, and 2.945 Å, indicate the integration of PAN nanofibers onto the GO surface, forming a crystalline composite structure^37,38^.

**Fig. 4:**
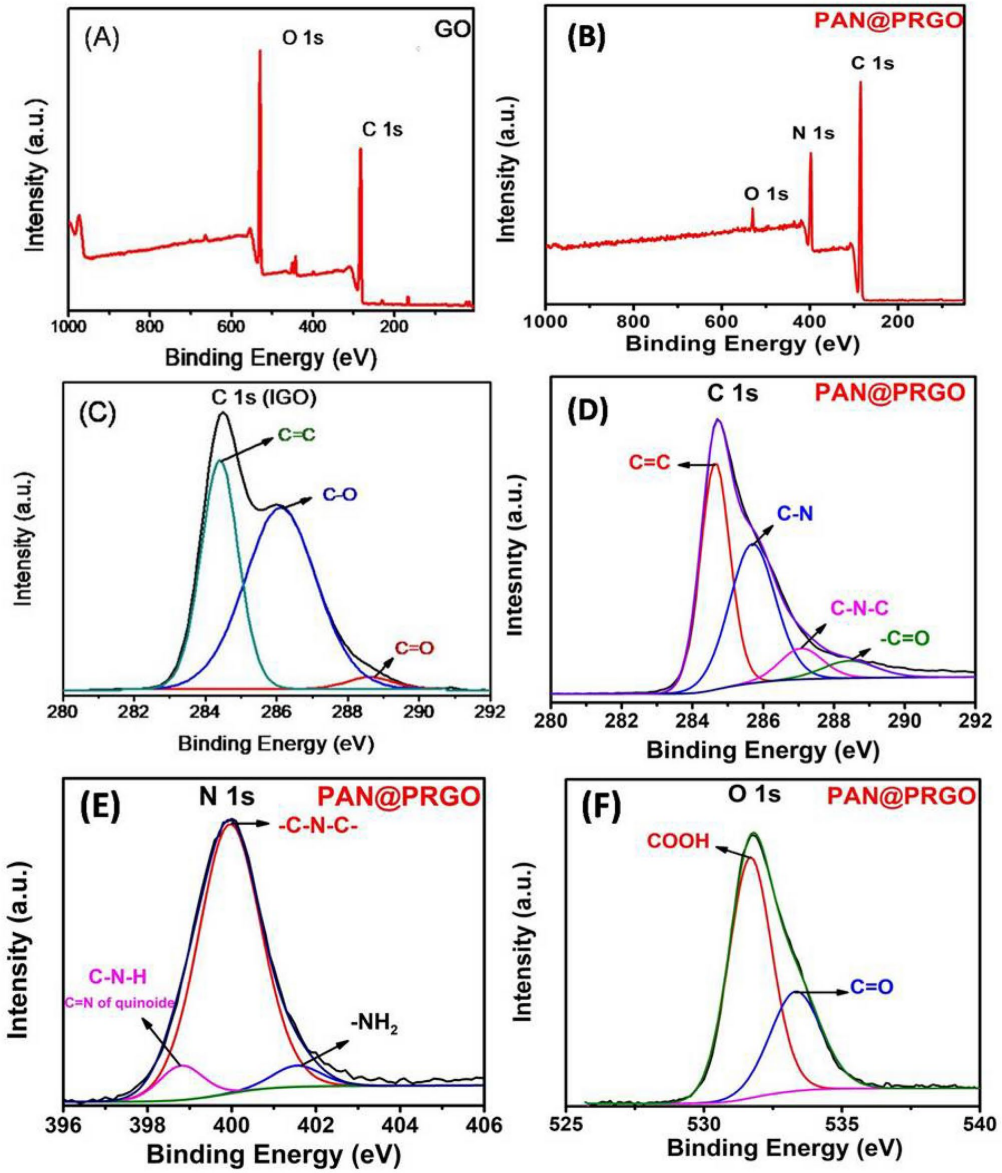
XPS spectra of (**A**) GO, and (**B**) PAN@PRGO; High resolution XPS spectra of (**C**) C 1s (GO), (**D**) C 1s (PAN@PRGO), (**E**) N 1s (PAN@PRGO), and (**F**) O 1s (PAN@PRGO).

The most common method for surface chemical analysis is XPS, which is used to look at individual elements in GO and PAN@PRGO composites. The formation of PAN@PRGO nanocomposite can also be confirmed by XPS, as shown in Fig. 4. In the XPS survey spectra of GO and PAN@PRGO (Fig. 4B), the intensity of the peak assigned to C1s significantly enhanced from GO to PAN@PRGO while the intensity of the O1 peak decreased from GO to PAN@PRGO. Additionally, a new peak of N1s is clearly observed in the XPS survey spectra of PAN@PRGO. These findings show that PAN nanoparticles were successfully grafted onto GO nanosheet surfaces. Three peaks with binding energies of 288.9 eV (C in C=O), 284.0 eV (Cin C=C, C–H), and 286.8 eV (C in C–O) were deconvoluted from the C1s high-resolution scan of GO^33^. The C1s high-resolution spectrum (Fig. 4D) related to PAN@PRGO was deconvulated to two peaks with bending energies of about 285.7 eV (C in C–N, C=N), 287.2 eV (C in C–N–C, C=N), and 288.7 eV (C in C=O)^33,36^.

The O1s high-resolution spectra of GO (Fig. 4F) were deconvoluted into two peaks at binding energies 531.7 and 533.3 eV, corresponding to (O in COOH) and (O in C=O), respectively. The high-resolution scan of the N1s peak of PAN@PRGO (Fig. 4E) was deconvoluted into three peaks at binding energies of 398.8 eV (N in C=N of quinoide), 399.9 eV (N in C–N–C), and another peak at 401.5 eV (N in –NH2)^33,36,39^. These observations confirm the successful grafting and polymerization of PAN nanoparticles onto the surface of GO nanosheets.

### Batch adsorption studies

#### Influence of pH

**Fig. 5:**
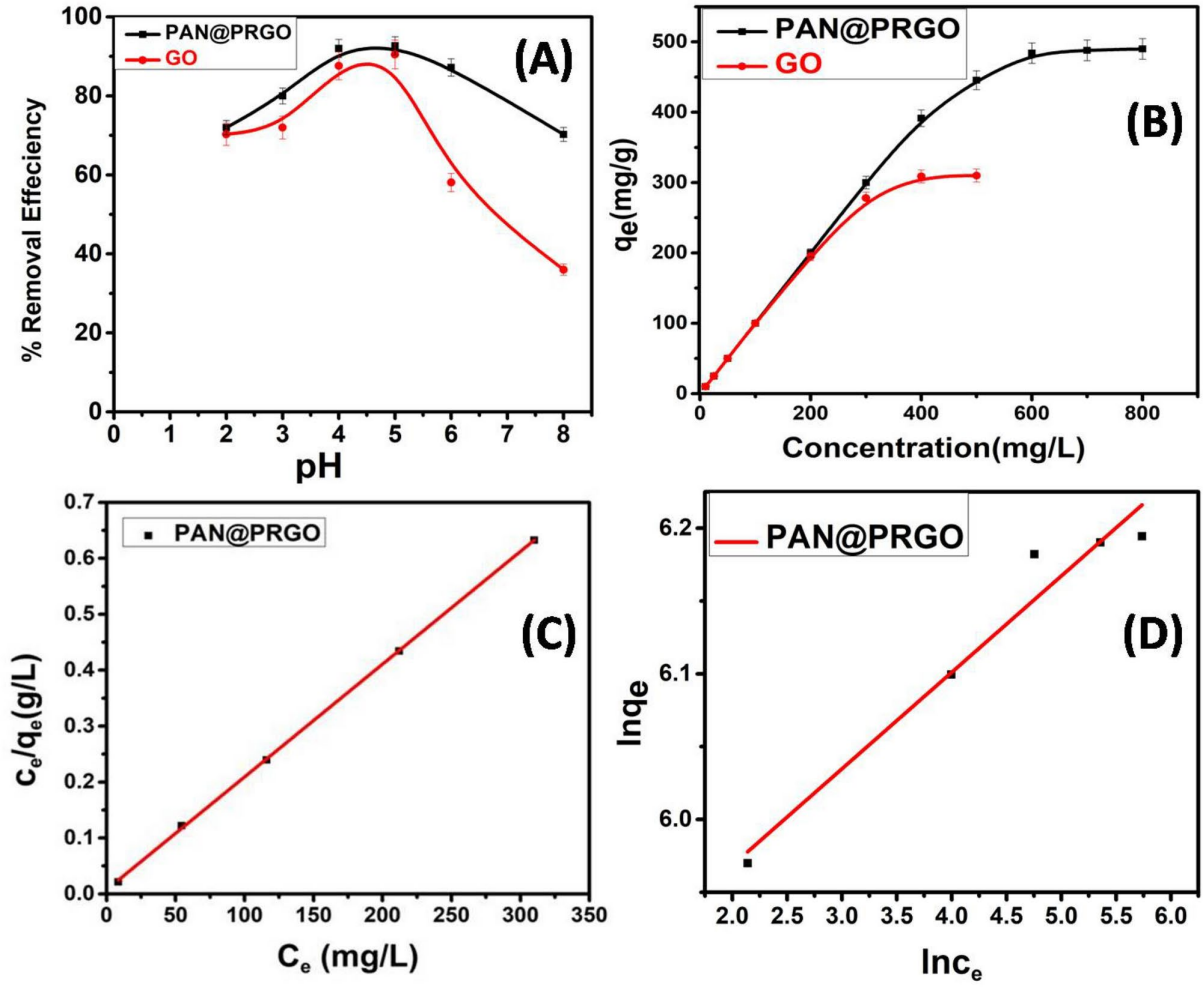
The adsorption efficiency of GO and PAN@PRGO composite as a function of (**A**) pH; (**B**) concentration; (**C**) Langmuir isotherm model and (**D**) Freundlich isotherm model.

The solution pH plays a crucial role in the adsorption process. The effect of pH on IC dye adsorption onto both GO and PAN@PRGO was investigated over a pH range of 2–8 using acetate buffer (pH 3–6) and phosphate buffer (pH 2, 8), as shown in Fig. 5A. The results demonstrated that the extraction efficiency of IC dye increased as the solution pH increased from 2.0 to 5.0, followed by a decline at higher pH values above 5. The optimal pH for IC dye adsorption onto GO and PAN@PRGO was found to be 5, with removal efficiencies of 90.48% and 92.66%, respectively. At low pH values, protonation of the functional groups on the adsorbents (COOH⁺2, O=C–NH⁺) increases the positive charge density on the adsorbent surface, thereby enhancing electrostatic attraction toward the negatively charged IC dye molecules and improving adsorption. However, at pH values above 5.5, adsorption efficiency decreases rapidly. This behavior can be attributed to the electrostatic repulsion between the negatively charged dye molecules and the increasingly negative surface of PAN@ PRGO^40–42^. Furthermore, this trend is consistent with the point of zero charge (pHpzc) of PAN@PRGO, which was determined to be 5.8 (Supporting Information, S2, Fig. S1)^43–45^. Below the pHpzc, the nitrogen- and oxygen-containing functional groups on PAN@PRGO are predominantly protonated, favoring electrostatic interactions with IC dye^46^. Conversely, at pH values above the pHpzc, the surface of PAN@PRGO becomes negatively charged, significantly reducing the attractive forces between the adsorbent and IC dye molecules, thereby lowering the adsorption efficiency. These findings are in agreement with previous studies^43–45,47,48^.

#### Impact of IC dye concentration

The influence of the initial concentration on the removal of IC dye using GO and PAN@PRGO was investigated at pH 5 and 25 °C by using 10.0 mg adsorbent dose and 10.0 mL of different IC dye concentrations (10.0 to 500.0 mg L^−1^) for GO and (10.0 to 800.0 mg L^−1^) for PANI-GO. Figure 5B showed that by increasing the concentration of IC dye (10.0 to 800.0 mg L^−1^), q_e_ increased from 10 to 490 mg g^−1^ for PAN@PRGO and from 10 to 310.0 mg/g for GO. This might be explained by the concentration gradient increasing the driving force, which improved mass movement^20,49,50^. Additionally, the findings also showed that the materials displayed 100% removal for concentrations up to 300 mg L^−1^for PAN@PRGO and 100.0 mg L^−1^for GO.

The Langmuir and Freundlich isotherm models models^51,52^, described by Eqs. (3), and (4) respectively, are very useful for describing the interaction nature between the adsorbent and adsorbate molecules. Langmuir model proposed monolayer and homogenous adsorption coverage. Whereas the Freundlich model based on heterogeneous active sites with multilayer adsorption.

3 $$\frac{{\text{C}}_{\text{e}}}{{\text{q}}_{\text{e}}}= \frac{1}{{\text{b Q}}_{0}} + \frac{{\text{C}}_{\text{e}}}{{\text{Q}}_{0}},$$

4 $${\text{lnq}}_{\text{e}}={\text{lnK}}_{\text{f}} + \frac{1}{\text{n}}\text{ ln}{\text{C}}_{\text{e}},$$

where q_e_ and q_max_ are the equilibrium and theoretical adsorption capacity of Langmuir monolayer respectively (mg g^−1^). C_e_ is the equilibrium concentration of dye (mg L^−1^). The parameter K_f_ (mg L^−1^) denotes the Freundlich constant related to adsorption capacity, and n represents the heterogeneity factor. The equilibrium adsorption constant is denoted as b (mg L^−1^) in the Langmuir isotherm model models^51,52^.

Figure 5C,D reveals the theoretical Freundlich and Langmuir adsorption isotherm which also describe the experimental data.

R_L_ parameter is given by Eq. (5)

5 $${\text{R}}_{\text{L}}= \frac{1}{1+{\text{bC}}_{0}}.$$

Table 1 shows that the correlation coefficient (R^2^) value for the Langmuir model is around 0.999, and the calculated q_m_ (495.04 mg g^−1^) is relatively close to the experimental q_exp._ (490.0 mg g^−1^). Thus, the IC dye adsorption is more in line with the Langmuir model, indicating a greater propensity for monolayer adsorption in the IC dye adsorption by PAN@PRGO composite. Additionally, Furthermore, R_L_ value range from 0 to 1 (Table 1), suggesting that the IC dye’s adsorption mechanism is favorable^53–55^.

**Table 1:** Isotherm variables for PAN@PRGO’s IC removal.

Dye	q_exp_. (mg g^−1^)	q_m_ (mg g^−1^)	Langmuir			Freundlich		
			b (mg^−1^ L)	R_L_	R^2^	K_f_ (mg^−1^ L)	1/n	R^2^
**IC**	490.0	495.04	0.269	0.005	0.999	342.4	0.066	0.945

#### Effect of adsorbent dosage

**Fig. 6:**
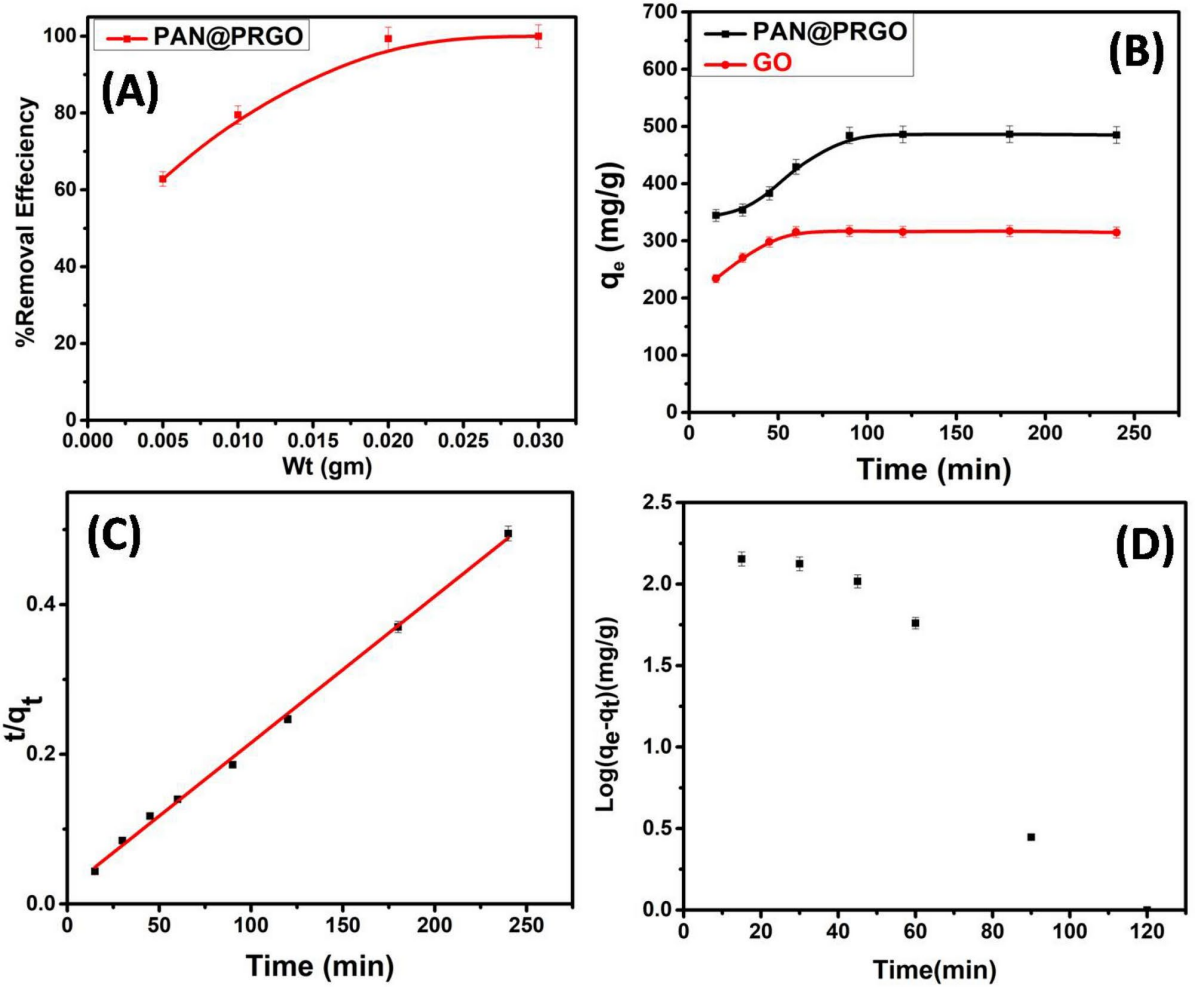
The adsorption efficiency of GO and PAN@PRGO composite as a function of (**A**) adsorbent dosage; (**B**) contact time; (**C**) Pseudo second order kinetic model and (**D**) Pseudo first order kinetic model.

The effect of adsorbent dosage (0.5 to 3.0 g L^−1^) on the removal of IC dye using PAN@PRGO was studied at pH 5, temperature 25 °C, IC concentration 600 mg L^−1^, and agitation time 120 min. Figure 6A showed that the removal efficiency of IC dye by PAN@PRGO increased from 62.82% to 100% by increasing the adsorbent dosage from 0.5 to 3.0 g L^−1^. This explained that at higher adsorbent dosages, more active sites are available, which boosted the extraction of higher amounts of IC dye^56^.

#### Effect of agitation time and adsorption kinetics

The contact time plays an important role in the investigation of the adsorption pathway until it reaches equilibrium and the rate of adsorption, as depicted in Fig. 6B. The results showed that the rate of adsorption of IC dye increased rapidly with increasing agitation time. In the first 60 min, more than 70.56% and 63.08% of IC dye were adsorbed onto PAN@PRGO and GO, respectively. This is due to the availability of a large number of available adsorption sites on the surface of the adsorbent. Additionally, the higher driving force boosted fast transfer of IC dye to the adsorbent surface^50^. The maximum adsorption of IC was achieved within 90 min at initial IC concentrations of 600 mg L^−1^ and 500 mg L^−1^ for PAN@PRGO and GO, respectively.

Two adsorption kinetic models (Fig. 6C,D) were utilized mainly to describe the adsorption process mechanism, pseudo-first-order and pseudo-second order expressed by Eqs. (6), and (7), respectively^20,57^.

6 $$\text{log}\left({\text{q}}_{\text{e}}- {\text{q}}_{\text{t}}\right)={\text{logq}}_{\text{e}} - \frac{{\text{K}}_{1}\text{ t}}{2.303},$$

7 $$\frac{\text{t}}{{\text{q}}_{\text{t}}}=\frac{1}{ {\text{K}}_{2}{{\text{q}}_{\text{e}}}^{2}} + \frac{\text{t}}{{\text{q}}_{\text{e}}},$$

where q_e_ and q_t_ are the adsorption uptake of IC dye at equilibrium and at time t (min) and k_2_ (g mol^−1^ min^−1^) and k_1_ (min^−1^) and are the rate constant for pseudo-second order and pseudo first order respectively.

**Fig. 7:**
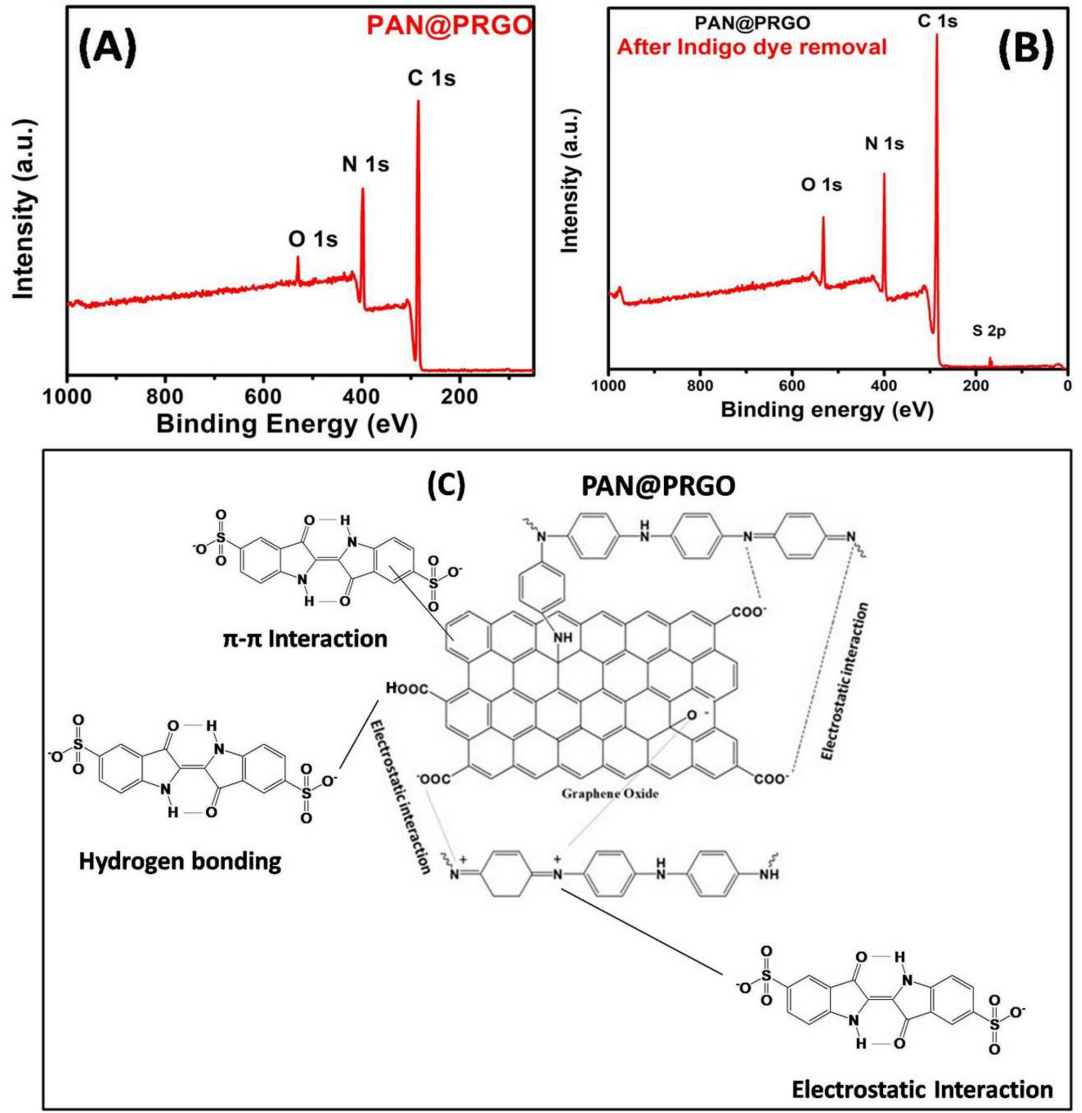
XPS survey spectra of PAN@PRGO before (**A**) and after (**B**) IC dye adsorption. (**C**) Proposed mechanism for IC removal by PAN@PRGO.

Table 2 summarized all the kinetic parameters calculated for adsorption of IC dye onto PAN@PRGO.The results demonstrated that the experimental values capacities (q_e.exp_) are in good agreement with calculated maximum uptake capacity using pseudo second order. Furthermore, the correlation coefficient (R^2^) of pseudo second order (0.999) is higher than those of pseudo first order as described in Fig. 6C,D. These confirmed that the adsorption of IC dye on PAN@PRGO fitted well with pseudo second order kinetic model through the sharing or exchanging of electrons between IC dye and PAN@PRGO composite. The interaction between PANG@PRGO and IC dye molecules was also confirmed by XPS analysis before and after dye adsorption. The XPS survey scans of PAN@PRGO before and after IC dye adsoprion showed two peaks, as shown in Fig. 7, which were caused by C 1s (285.7 eV), O 1s (532.9 eV), and N 1s (400.9 eV). Additionally, the presence of indigo carmine dye was indicated by an extra peak in the XPS survey spectra of PAN@PRGO---IC caused by S 2p (167 eV). These findings support the concept that the indigo carmine attaches to the PAN@PRGO via hydrogen bonding between the IC dye molecules and the –C=NH, –NH, and –C=O groups of the PAN@PRGO as well as electrostatic attraction (Fig. 7C).

**Table 2:** Parameters of PSO and PFO models for the adsorption of IC dye onto PAN@PRGO.

Dye	q_e,exp._ (mg g^−1^)	PSO			PFO		
		q_e,cal._ (mg g^−1^)	K_2_ (min g mol^−1^)	R^2^	q_e,cal_ (mg g^−1^)	K_1_ (min^−1^)	R^2^
IC	490.0	510.2	0.0002	0.997	16.6	0.053	0.901

#### Influence of temperature and thermodynamic studies

The effect of temperature parameter on the adsorption capacity of IC dye using PAN@PRGO is presented in Fig. S2A. It utilized that by increasing the temperature from 298 to 328 K, the maximum adsorption capacities of IC dye onto PAN@PRGO adsorbent increased from 490.0 to 530.98 mg g^−1^ at T = 318 K and from 490 to 590.93 mg.g^−1^ at T = 328 K. This may be attributed to the increase in the kinetic energy and mobility of IC dye molecules with the temperature^2^.

The thermodynamic parameters (ΔH°, ΔG°, and ΔS°) were determined by Eqs. (8), (9), and (10).

8 $${\Delta \text{G}}^{^\circ }= {\Delta \text{H}}^{^\circ } - {\text{T }\Delta \text{S}}^{^\circ }.$$

Van’t Hoff equation

9 $${\text{Lnk}}_{\text{d}}= \frac{\Delta \text{S}^\circ }{\text{R}}- \frac{\Delta \text{H}^\circ }{\text{RT}},$$

10 $${\text{K}}_{\text{d}}=\frac{{\text{q}}_{\text{e}}}{{\text{c}}_{\text{e}}} *\uprho .$$

ΔH°, ΔG°, and ΔS° stand for the standard enthalpy change, standard free energy change, and standard entropy change, respectively^2^. The symbols Kd, T, and R stand for the adsorption coefficient, temperature (k), and gas constant (8.314 J mol^−1^ K^−1^), respectively^50^. To create a dimensionless Kd, use ρ = 1000 g/L. The ΔS° and ΔH° values were calculated using the intercept and slope of the plot of ln Kd vs 1/T (Fig. S2B). The computed thermodynamic functions are summarized in Table 3. An rise in the solid/solute interface’s randomness throughout the adsorption process is examined by the positive entropy. A spontaneous adsorption process is suggested by a decrease in the negative value of free energy (ΔG°), while an endothermic adsorption process is indicated by a positive value of enthalpy (ΔH°).

**Table 3:** Thermodynamic variables for the removal of IC dye onto PAN@PRGO at various temperatures.

Metal concentration (mg/L)	∆H° (kJ/mol)	∆S° (kJ/mol)	∆G° (kJ/mol)		
			298 K	308 K	323 K
500.0	117.86	0.468	− 21.6	− 26.3	− 33.3
600.0	37.709	0.194	− 20.1	− 22.0	− 24.9
700.0	25.678	0.149	− 18.7	− 20.2	− 22.4

#### Reusability of PAN@PRGO adsorbent

**Fig. 8:**
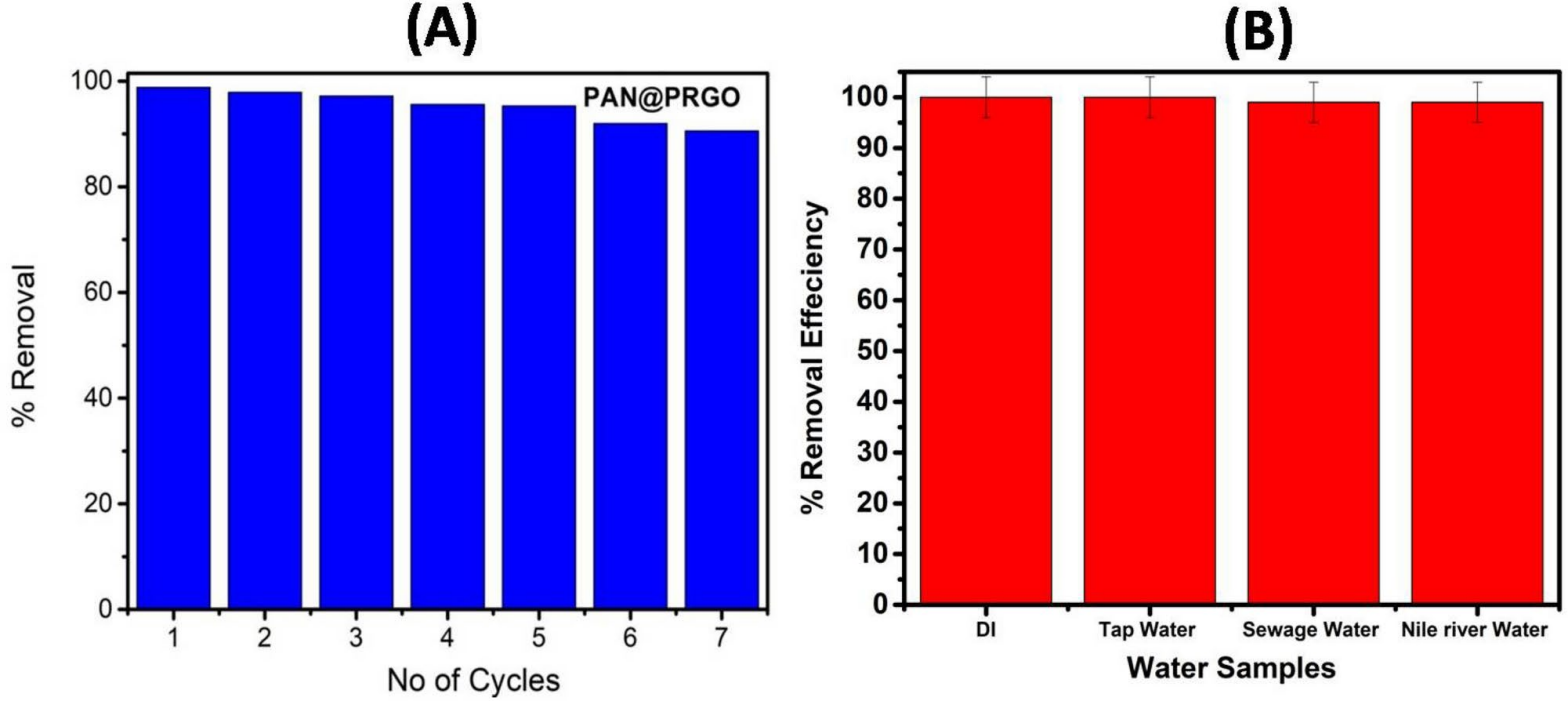
(**A**) Reusability of PAN@PRGO composite. (**B**) Real sample analysis.

The ability of an adsorbent to be reused is one of its most crucial characteristics, as it affects both its long-term and economical viability in industrial applications. Eluent (desorbing agent) 0.1 M NaOH was used, as explained in the experimental section. The PAN@PRGO adsorbent’s regeneration and reusability across seven cycles were described in detail in Fig. 8. After seven cycles, the findings showed that the adsorption efficiency was approximately 93.0%. These results offer important information for the creation of an adsorbent PAN@PRGO with excellent stability, high efficiency, and low cost effectiveness to address dye pollution.

#### Application in real water samples

To evaluate the performance of PAN@PRGO under optimal conditions, adsorption experiments were conducted using different real water sources, including tap water, Nile water, and sewage water (Fig. 8B). A reference solution containing 100 mg L^−1^ of IC was prepared for comparison. Water samples were collected from municipal tap water, a designated section of the Nile River, and a local sewage treatment facility for laboratory analysis. The adsorption studies demonstrated that PAN@PRGO maintained a removal efficiency exceeding 99.0% across all tested water matrices (Fig. 8B). Despite the presence of various dissolved minerals and organic pollutants in river and wastewater samples, the adsorbent exhibited consistent performance. All batch adsorption experiments were performed in triplicate, yielding relative standard deviation (RSD) values below 4%, ensuring data reliability. These findings underscore the potential applicability of PAN@PRGO for efficient dye removal in diverse environmental water sources.

#### Comparison with literature

Table 4 provides a comparison of the q_max_ values for IC dye adsorption onto PAN@PRGO, GO, and other materials reported in the literature. Both PAN@PRGO and GO demonstrated remarkable adsorption capacities for removing IC dye from aqueous solutions.

**Table 4:** Comparison of the sorption capacities of various adsorbents for IC dye.

Pollutant	Adsorbents	q_e max_ (mg g^−1^)	References
IC	PAN@PRGO	490.0	This work
	GO	317.25	This work
	AlCuFe-LDH/C nanocomposite	26.56	^58^
	SBA-15/PANI/PPy composite	384.61	^59^
	alumina beads	126.0	^60^
	Polyethylene Glycol-Modified Hydroxyapatite	129.0	^61^
	CuFe_2_O_4_-NPs (CFN)	57.4	^62^
	Graphene nano dots (GNDs)	39.07	^63^
	PCNTs	93.0	^25^
	5% (MgOBi_2_)	126.0	^64^

## Conclusions

The present study elucidates the successful synthesis of PAN@PRGO nanocomposite via in situ polymerization of aniline (AN) onto the surface of GO nanosheets. The PAN@PRGO was fully characterized using different analytical techniques FTIR, XPS, XRD and SEM. The prepared PAN@PRGO nanocomposite exhibited an effective efficiency for the removal of IC dye (490.0 mg/g) via electrostatic attraction and hydrogen bonding between –C=NH, –NH and C=O groups of PAN@PRGO and IC dye molecule. The optimum conditions for the removal of IC dye using PAN@PRGO were pH 5, 1 g/L of adsorbent, and 90 min of room temperature agitation. In addition, the pseudo-second-order model and the Langmuir isotherm model for the adsorption process are followed, suggesting a monolayer adsorption mechanism. Additionally, the outcomes demonstrated a little reduction in adsorption efficiency during seven cycles, suggesting that PAN@PRGO composite might be used as superior adsorbent for the remediation of industrial effluents containing organic dyes.

## Supplementary Information

Supplementary Information.

## Data Availability

All data generated or analysed during this study are included in this published article [and its supplementary information files].
