# Supplementary material for: Enhanced removal of indigo carmine dye from aqueous solutions using polyaniline modified partially reduced graphene oxide composite
Source: Sci Rep. 2025 May 3;15:15555. doi: 10.1038/s41598-025-98115-8 (PMC12049445; doi:10.1038/s41598-025-98115-8)
Supplement: Supplementary file 1 — Supplementary Information. [file 41598_2025_98115_MOESM1_ESM.doc]

**Enhanced Removal of Indigo Carmine Dye from Aqueous Solutions Using Polyaniline Modified Partially Reduced Graphene Oxide Composite**

Saadia M. Waly^1^, Ahmad M. El-Wakil^1^, Mohamed M. Waly^1,2^, Weam M. Abou El-Maaty^1^, and Fathi S. Awad^1,2*^

^1^Chemistry Department, Faculty of Science, Mansoura University, Mansoura 35516, Egypt.

^2^ Chemistry Department, Faculty of Science, New Mansoura University, New Mansoura 35712, Egypt.

**Supporting Information**

**S1: Characterization**

The GO nanosheets and PAN@PRGO nanocomposite were characterized using a diversity of analytical techniques. Fourier transform infrared (FTIR) spectra were recorded using a Nicolet-Nexus 670 spectrometer equipped with a diamond ATR accessory. The measurement employed a resolution of 4 cm⁻¹ and 32 scans. The crystal structure of the sample was analyzed using powder X-ray diffraction (XRD) on a PANalytical MPD X’Pert PRO diffractometer. The measurements employed 45 kV, 40 mA Ni-filtered Cu Kα1 radiation at room temperature. X-ray photoelectron spectra (XPS) were obtained on a Thermo-Fisher ESCALAB 250 spectrometer equipped with a micro-focused, monochromated Al Kα X-ray source operating at 15 kV and a double-focusing, full 180° spherical sector electron analyzer. Scanning electron microscopy (SEM): A Hitachi SU-70 field-SEM with an energy of 5.0 kV was used to take the SEM images. Transmission electron microscopy (TEM): A JEOL JEM1400 TEM was used to obtain the TEM images at 100 kV.

S2: **Determination of pHpzc**

The point of zero charge (pH_PZC_) was determined using the pH drift method. A series of vials were prepared, each containing 5.0 mg of PAN@PRGO and 10 mL of a 0.1 M NaCl solution, with initial pH values ranging from 2.0 to 12.0. These mixtures were agitated at 200 rpm at room temperature (25.0 ± 1.0 °C) for 24 hours. The change in pH (ΔpH = pH_f_ – pH_i_) was plotted against the initial pH (pH_i_), and the pHpzc was derived from the intersection with the x-axis(Lawtae & Tangsathitkulchai, 2021). Identifying the pHpzc of an adsorbent is essential for selecting an optimal solution pH to enhance the adsorption process. When the pH is below the pHpzc, the adsorbent surface is positively charged, favoring the adsorption of anionic dyes (IC). The results are illustrated in Figure S1. The pHpzc value of the PAN@PRGO is 5.8.


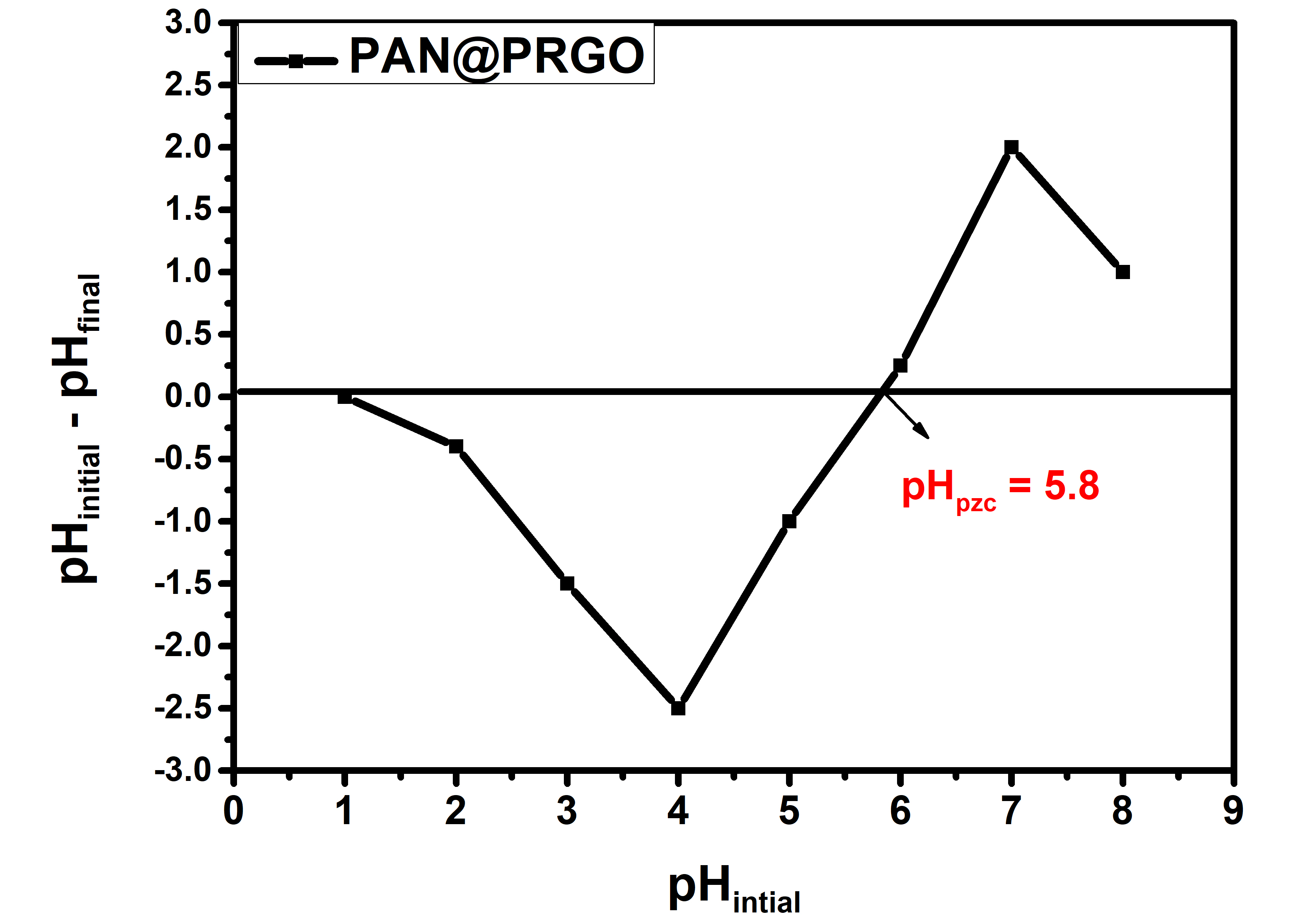


Figure S1: Determination of pHpzc for PAN@PRGO.


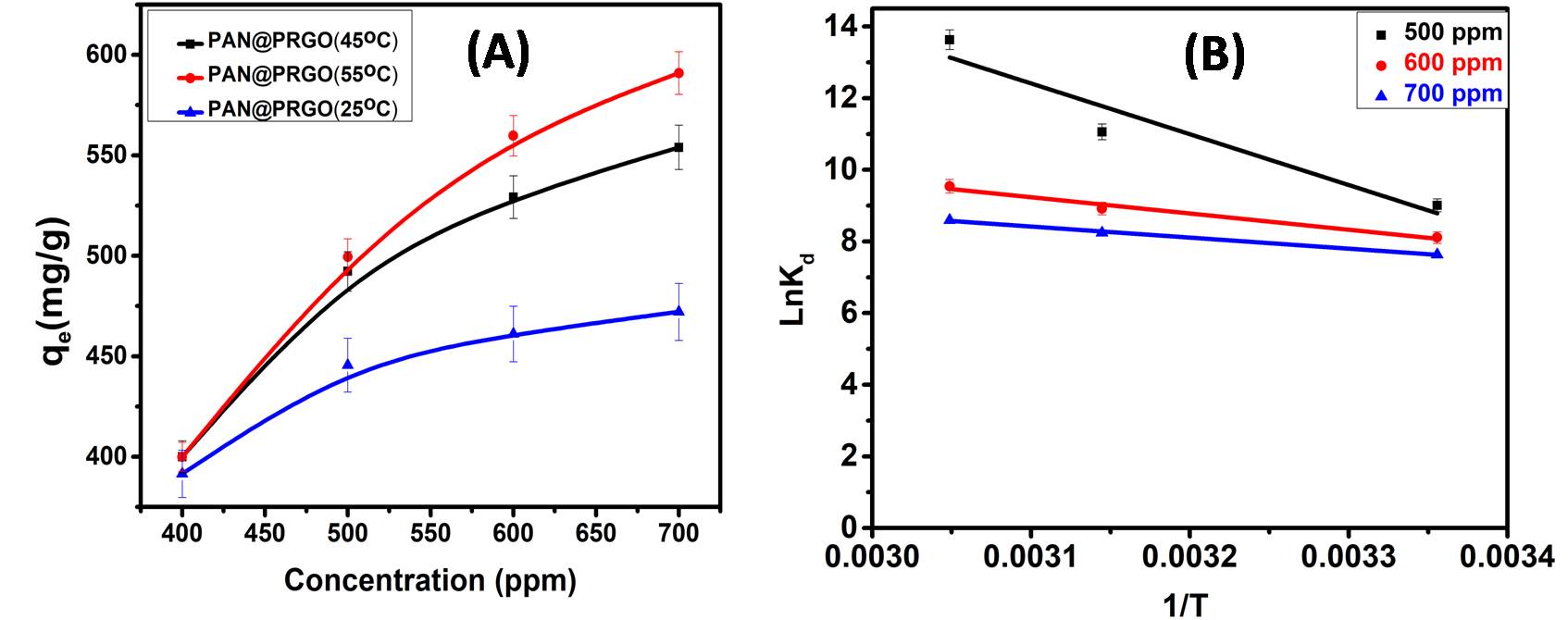


Figure S2: (A) The adsorption capacity of PAN@PRGO as a function of temperature; (B) The plots of lnKd versus T^−1^ for estimations of thermodynamic parameters of the adsorption process of IC dye on PAN@PRGO.
